# BRAF, MEK, and EGFR Triplet Inhibitors as Salvage Therapy in *BRAF*-Mutated Metastatic Colorectal Cancer—A Case Series Study *Target Therapy of* BRAF-*Mutated mCRC*

**DOI:** 10.3390/medicina57121339

**Published:** 2021-12-07

**Authors:** Jen-Hao Yeh, Hsiang-Lin Tsai, Yen-Cheng Chen, Ching-Chun Li, Ching-Wen Huang, Tsung-Kun Chang, Wei-Chih Su, Po-Jung Chen, Yu-Peng Liu, Jaw-Yuan Wang

**Affiliations:** 1 Graduate Institute of Clinical Medicine, College of Medicine, Kaohsiung Medical University, Kaohsiung 80708, Taiwan; b9202078@gmail.com (J.-H.Y.); ypliu@kmu.edu.tw (Y.-P.L.); 2 Division of Gastroenterology and Hepatology, Department of Internal Medicine, E-DA Dachang Hospital, Kaohsiung 80794, Taiwan; 3 Department of Medical technology, College of Medicine, I-Shou University, Kaohsiung 82445, Taiwan; 4 Division of Gastroenterology and Hepatology, Department of Internal Medicine, E-DA Hospital, Kaohsiung 82445, Taiwan; 5 Division of Colorectal Surgery, Department of Surgery, Kaohsiung Medical University Hospital, Kaohsiung Medical University, Kaohsiung 80708, Taiwan; chunpin870132@yahoo.com.tw (H.-L.T.); googoogi05@gmail.com (Y.-C.C.); dobird05@yahoo.com.tw (C.-C.L.); baseball5824@yahoo.com.tw (C.-W.H.); tsungkunchang@gmail.com (T.-K.C.); lake0126@yahoo.com.tw (W.-C.S.); glaudiotennis@gmail.com (P.-J.C.); 6 Department of Surgery, Faculty of Medicine, College of Medicine, Kaohsiung Medical University, Kaohsiung 80708, Taiwan; 7 Center for Cancer Research, Kaohsiung Medical University, Kaohsiung 80708, Taiwan; 8 Center for Liquid biopsy and Cohort Research, Kaohsiung Medical University, Kaohsiung 80708, Taiwan; 9 Pingtung Hospital, Ministry of Health and Welfare, Pingtung 90054, Taiwan

**Keywords:** metastatic colorectal cancer, *BRAF* mutation, triple target therapy

## Abstract

*Backgroundand objectives*: Patients with *BRAF*-mutated metastatic colorectal cancer have considerably poorer responses to conventional systemic treatment. The real-world effects of triplet therapy with BRAF, mitogen-activated protein kinase kinase, and epidermal growth factor receptor inhibitors in Asia have not been well-reported. *Materials and Methods*: This single-center case series included patients with *BRAF*-mutated metastatic colorectal cancer undergoing triplet therapy after failure of prior systemic treatment from 2016 to 2020. The primary outcome was progression-free survival, and secondary outcomes were overall survival, response rate, disease control rate, and adverse events. *Results*: Nine eligible patients with *BRAF*-mutated metastatic colorectal cancer receiving triplet therapy were enrolled, with a median follow-up time of 14.5 months (range, 1–26). Most patients (88.8%) had two or more prior systemic treatments, and the triplet regimen was mainly dabrafenib, trametinib, and panitumumab. The overall response rate and disease control rate were 11.1% and 33.3%, respectively. Median progression-free survival and overall survival were 2.9 and 7.4 months, respectively, and a trend toward better overall survival was found with left-sided metastatic colorectal cancer compared with right-sided disease (9.2 vs. 6.9 months, *p* = 0.093). Adverse events were mostly Grade 1–2, including nausea, hypertension, gastrointestinal symptoms, and skin disorders. *Conclusions*: In this single-center case series, triplet therapy with BRAF, mitogen-activated protein kinase kinase, and epidermal growth factor receptor inhibitors in *BRAF*-mutated metastatic colorectal cancer had an acceptable safety profile and reasonable efficacy.

## 1. Introduction

Cases of metastatic colorectal cancer (mCRC) comprise approximately one-fourth of all colorectal cancer (CRC) cases at initial diagnosis, and an additional 20% of CRC patients may also present subsequent metachronous metastasis despite treatment [1,2]. Progress has been made in various treatment strategies, including surgery, cytotoxic chemotherapy, target therapy, and immunotherapy. RAS wild type mCRC is still a treatment challenge, especially when other resistant gene alterations are present.

Along with RAS [3,4] and microsatellite instability [5,6], the BRAF V600E mutation [7,8] is a well-known biomarker that has an impact on mCRC survival and may affect the response of systemic and targeted therapies. Although the BRAF mutation is only detected in 5%–10% of all cases, mCRC patients who are microsatellite-stable with the BRAF V600E mutation have worse survival and response to anti-epidermal growth factor receptor (EGFR) agents [9,10]. However, resistance to anti-EGFR agents may be overcome with BRAF inhibitors [11,12], which may be beneficial in patients with progressive mCRC after the failure of first-line treatment.

The combination of a BRAF inhibitor and anti-EGFR agent, with and without a mitogen-activated protein kinase kinase (MEK) inhibitor, has been evaluated in several studies as a promising regimen for mCRC after first-line standard treatment [11,13,14]. The phase III BEACON trial demonstrated that the triplet regimen, which consists of a BRAF inhibitor, anti-EGFR agent, and MEK inhibitor, significantly improved overall survival (OS) and progression-free survival (PFS) compared with the control group (chemotherapy plus anti-EGFR agent) [11]. Another ongoing single-arm trial (ANCHOR CRC, a phase II study of first-line triple therapy with cetuximab, encorafenib, and binimetinib) also showed a favorable response rate [15]. However, real-world data on the triplet regimen as a later line of systemic treatment in Asian patients, is still lacking due to the scarcity of such patients. Thus, this case series aimed to report the clinical outcomes and safety of triplet therapy in mCRC patients with BRAF V600E mutations after the failure of at least first-line chemotherapy.

## 2. Methods

### 2.1. Patient Eligibility

This case series was a single-center study conducted at our hospital. Eligible cases were identified through medical chart review from April 2016 to April 2020. Patients were included if they met all the following criteria: (1) recurrence or progressive disease after first-line chemotherapy plus target therapy, with or without surgery; (2) at least one metastatic focus found in an imaging study; (3) pathologic examination of the tumor specimen revealing a BRAF V600E mutation; and (4) receiving triplet therapy as the second or later line of systemic treatment. Eligible cases were enrolled for this study until April 2020. This study was approved by the institutional review board of our hospital [KMUHIRB-2012-03-02(II)].

### 2.2. Analysis of BRAF Mutation, RAS Mutation, and Status of Microsatellite Stability

BRAF V600E mutation analysis was performed using direct deoxyribonucleic acid (DNA) sequencing from formalin-fixed, paraffin-embedded CRC tissue samples according to our previous study [16]. After deparaffinization and air-drying, DNA was isolated using the proteinase K and QIAamp DNA Micro Kit (QIAGEN). A high-resolution melting analysis was undertaken using the LightCycler 480 System Gene Scanning Assay. The primers used, which were specific for the BRAF V600E mutation, were designed using Primer3 free software. The forward and reverse primer sequences were 5′-CATAATGCTTGCTCTGATAGGAAA-3′ and 5′-TCAGCACATCTCAGGGCCAAA-3′, respectively. All the primers were produced with standard molecular biology quality (Protech Technology Enterprise Co., Ltd., Taipei, Taiwan). RAS mutations were identified through direct DNA sequencing, the procedure for which was described in detail in our previous study [17]. Both KRAS and NRAS mutation statuses were examined in the patients. The presence of a deficient mismatch repair gene (dMMR) was determined by immunohistochemical staining of CRC tissue specimens. Loss of at least one mismatch repair protein (MLH-1, MSH-2, MSH-6, or PMS-2) was deemed indicative of the presence of dMMR [18].

### 2.3. Systemic Treatment and Outcome Assessment

In this case series, all eligible patients received the triplet regimen, which comprised the BRAF inhibitor dabrafenib (Novartis Pharmaceuticals, Basel, Switzerland), the MEK inhibitor trametinib (Novartis Pharmaceuticals, Basel, Switzerland), and the anti-EGFR agent panitumumab (Amgen Inc., Thousand Oaks, CA, USA) or cetuximab (Merck Sharp & Dohme Corp., Kenilworth, NJ, USA), after progressive disease was treated with at least second-line systemic treatment, including chemotherapy plus target therapy. The dosages were as follows: dabrafenib, 150 mg orally, twice per day; trametinib, 2 mg orally, once per day; panitumumab, 6 mg/kg every two weeks intravenously; and cetuximab, 400 mg/m^2^ loading, then 500 mg/m^2^ biweekly, intravenously. The patients attended regular follow-up visits at outpatient clinics every 2 weeks to evaluate symptoms and adverse events by the visiting staff and study nurses. When the patients were hospitalized for treatment or any other reason, the visiting staff and study nurses would be informed to allow assessment. The adverse events were recorded and graded during each cycle based on the National Cancer Institute Common Terminology Criteria for Adverse Events (Version 4.3; http://ctep.cancer.gov/reporting/ctc.html). Symptomatic treatments were provided for milder (grade 1-2) adverse events without interruption of systemic therapy, and the triplet therapy would be temporarily withheld for more severe adverse events (grade 3). Triplet therapy was only resumed if the adverse events were not life-threatening, and the patient got substantial improvement. The treatment response was typically assessed after 8–12 weeks of treatment by computed tomography, magnetic resonance imaging, or positron emission tomography according to the criteria of the Response Evaluation Criteria in Solid Tumors (RECIST; version 1.1) [19]. The median follow-up period was 14.5 (range, 1–26) months.

The primary outcome of this study was PFS, and secondary outcomes were OS, response rate (RR), disease control rate (DCR), and adverse events (AEs) of treatment. PFS was defined as the time from the initiation of the triplet regimen to the first radiological progression or tumor-related death, whichever came first. OS was defined as the time from the initiation of the triplet regimen to death due to any cause. DCR was represented as the percentage of patients with complete response, partial response, or stable disease as their best response.

### 2.4. Statistics

SPSS (Version 20.0; SPSS, Chicago, IL, USA) was used for all data analyses. The continuous variables were compared with Wilcoxon’s signed-rank test, and categorical variables were compared using the Chi-square test. The Kaplan–Meier method was used to calculate PFS and OS, and a log-rank test was used to compare time-to-event distributions by clinical and molecular factors. Statistical significance was set at *p* < 0.05.

## 3. Results

This section may be divided by subheadings. It should provide a concise and precise description of the experimental results, their interpretation, as well as the experimental conclusions that can be drawn.

### 3.1. Baseline Characteristics of Included Patients

This case series included nine patients (4 had primary tumors on the right side: 2 in ascending colon and 2 in transverse colon; and 5 on the left side: 3 in descending colon cancer and 2 in sigmoid colon) with BRAF V600E-mutated mCRC who underwent triplet therapy. Their baseline characteristics are displayed in Table 1. All patients had tumors with wild-type KRAS/NRAS and moderate to poor differentiation. dMMR was noted in two of the six analyzed patients. Most patients received panitumumab, dabrafenib, and trametinib as the triplet regimen, but one patient used cetuximab instead of panitumumab. In addition, triplet therapy was exclusively used as third-line or later treatment in all but one patient, for whom the therapy was initiated after the failure of first-line therapy. Most patients (77.7%) had liver metastases, and in nearly half of them (44.4%), at least three organs were involved at the time of treatment. No significant differences in baseline characteristics were observed between left-sided and right-sided mCRC.

### 3.2. Response Rate and Survival Analysis

**Figure 1:**
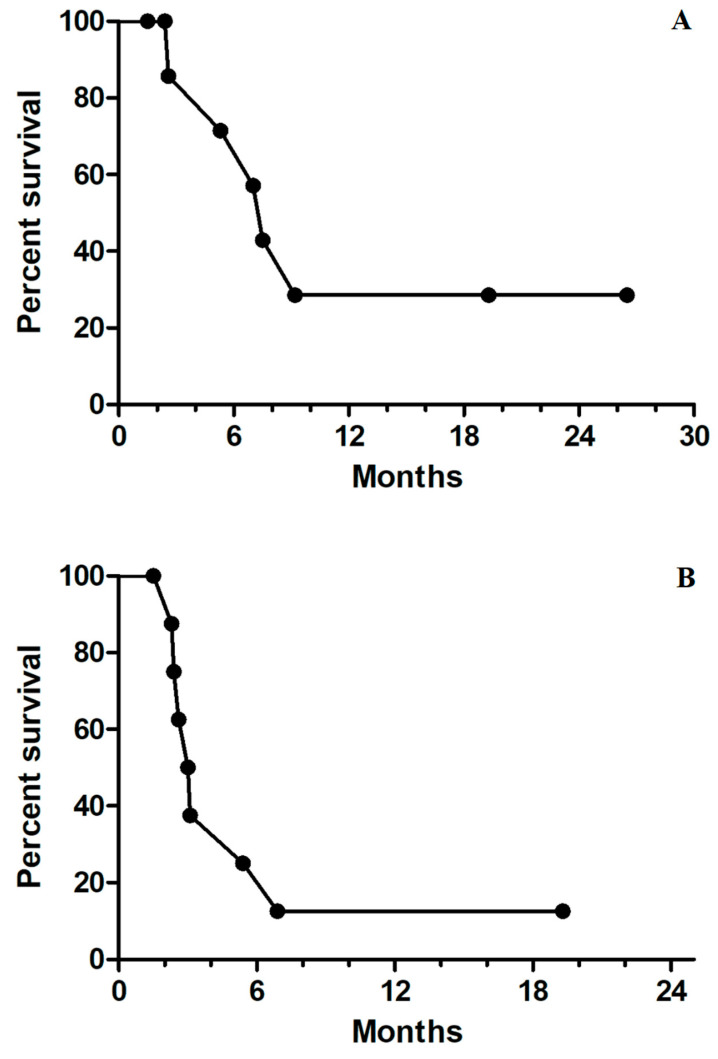
(**A**) Kaplan–Meier survival curves for median progression-free survival of 2.9 months for all nine patients; (**B**) Kaplan–Meier survival curves for median overall survival of 7.4 months for all nine patients.

**Figure 2:**
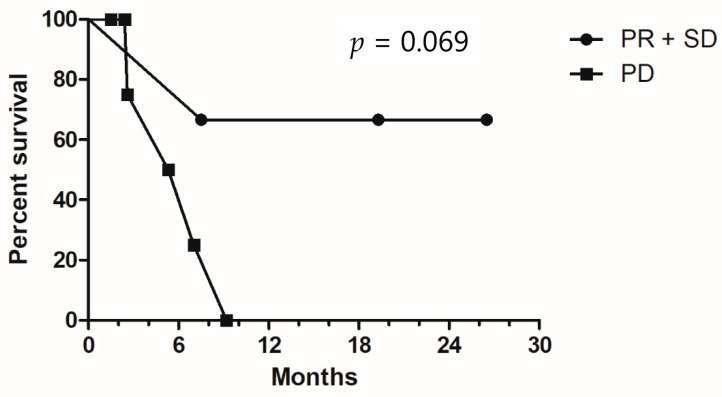
Kaplan–Meier survival curves for overall survival, stratified by disease control status. PR, partial response; SD, stable disease; PD, progressive disease.

Among the patients who underwent triplet therapy, only one patient had a partial response, and another two had stable disease (Table 2). All other patients had disease progression despite treatment (RR, 11.1%; DCR, 33.3%). The median PFS and OS were 2.9 months and 7.4 months, respectively (Figure 1A,B). No specific clinical or molecular factors were found to be significantly associated with favorable DCR or OS. However, a trend toward improved OS was found in left-sided mCRC compared with right-sided disease (9.2 vs. 6.9 months, *p* = 0.093) and patients with disease control. Median survival was not reached for patients with partial response or stable disease, and the median OS was 5.2 months for those with progressive disease (*p* = 0.069, Figure 2). In one patient with initial partial response after triplet therapy, PFS time persisted for 26 months until the last follow-up. In two patients with stable disease after triplet therapy, one had disease progression 3 months later and died, and the other patient achieved a PFS of 19 months without further systemic treatment.

### 3.3. Adverse Events

The adverse events in patients who received triplet therapy are summarized in Table 3. Triplet therapy was generally well-tolerated, and most adverse events were Grades 1–2. The most frequent adverse events were liver function abnormality (66.6%), hypertension (66.6%), and dermatitis (66.6%), followed by nausea (44.4%) and skin rash (44.4%). The most frequent severe events (Grade 3) were nausea (22%), hypertension (22%), dermatitis (22%), and diarrhea (11%). Of note, one patient developed blurred vision during the second month of triplet therapy, which gradually improved following the completion of systemic treatment and conservative management. No patient experienced grade 4 adverse events.

## 4. Discussion

In this case series, we demonstrated the real-world experience of using triplet therapy for BRAF-mutated mCRC as later lines of salvage therapy in Asian patients. Our findings suggest that triplet therapy appears to be well-tolerated and patients with initial disease control and longer PFS might gain considerable survival benefit, although most patients in our study still experienced disease progression.

The clinical efficacy of triplet therapy in BRAF-mutated mCRC has been demonstrated in two large clinical trials by Corcoran et al. [14] and Kopetz et al. (the BEACON trial) [11], and further trials are ongoing [15,20]. The trial by Corcoran et al. was a phase I trial using dabrafenib, panitumumab, and trametinib as triplet therapy, which was in line with our study’s regimen. Triplet therapy resulted in a 21% RR, and median PFS and OS were 4.2 and 9.1 months, respectively. Nevertheless, the BEACON trial showed that triplet therapy (encorafenib, binimetinib, and cetuximab) had a 26% RR, and median PFS and OS were 4.3 months and 9.0 months, respectively; by contrast, the control group had only a 2% RR, and median PFS and OS were 1.5 months and 5.4 months, respectively. Of note, these two trials included a considerable portion of patients who failed to respond to first-line treatment; by contrast, in the current study, most patients previously underwent at least second-line systemic treatment. Although a direct comparison between our study and those mentioned above was not possible, the RR and survival in the current study seem acceptable.

Another compelling question is whether primary tumor location affects the outcome in BRAF-mutated mCRC treated with triplet therapy. Although the role of tumor location in the prognosis of BRAF-mutated mCRC remains controversial [16,21], it may have some impact with the concomitant use of target therapy such as bevacizumab or cetuximab [22]. Several studies have demonstrated that first-line bevacizumab plus chemotherapy resulted in a superior prognosis for right-sided BRAF-mutated mCRC [16,23,24,25]. Conversely, left-sided mCRC had more favorable outcomes when treated with anti-EGFR agents than did right-sided tumors [22,26], which was consistent with our observation. Further studies exploring the impact of the primary tumor side on the prognostic outcomes of BRAF-mutated mCRC treated with anti-EGFR agents may be quite valuable.

Several factors may have contributed to the discrepancies related to treatment response and survival between this study and others. First, our study included mostly patients who underwent two or more prior systemic treatments, a factor that has been found to be associated with a worse RR [27,28]. Second, the difference between clinical trials and real-world practice may lead to some bias in objective evaluation. Other clinical factors, such as the presence of dMMR [29], differences in ethnicity, and different regimens, as well as the genetic alteration patterns of the BRAF mutation [30], might also influence the outcomes. These factors warrant a higher case enrollment and detailed analysis to clarify the best candidates for triplet therapy as salvage therapy among BRAF-mutated mCRC patients.

Regarding adverse events with triplet therapy, the most common AEs, including gastrointestinal and dermatologic disorders, were similar to those in previous clinical trials [11,14]. Our study did not observe any cases that required dose escalation or discontinuation due to side effects; one patient developed blurred vision, but the cycle of triplet therapy was maintained after two weeks until this symptom subsided. A previous study demonstrated that MEK inhibitors can induce retinopathy [31]. The onset is typically rapid in the first week of treatment but often resolves gradually, even without drug interruption. Thus, although this unique adverse event must be carefully monitored, it typically does not cause a serious sequela.

To the best of our knowledge, this is the first real-world study of triplet therapy in BRAF-mutated mCRC in Asian patients. In this study, we demonstrated an acceptable safety profile for triplet therapy, and we expect prolonged survival when initial disease control is obtained, even with two or more failures of prior systemic treatments. However, the limited case number precluded a robust subgroup analysis, and more data are necessary to explore the predictive factors of the prognosis of triplet therapy for BRAF-mutated mCRC in real-world practice.

In summary, this single-center case series demonstrated that triplet therapy with BRAF and MEK inhibitors and an anti-EGFR agent had an acceptable safety profile and reasonable efficacy for BRAF-mutated mCRC. Further studies enrolling more patients are needed to identify potential treatment responses and improve the efficacy of the treatment regimen.

## Figures and Tables

**Table 1:** Baseline characteristics of all included patients with *BRAF*-mutated mCRC receiving triplet therapy, stratified by tumor sidedness.

Characteristic	All Patients (*N* = 9)	Right Side Tumor (*N* = 4)	Left Side Tumor (*N* = 5)	*p* Value
Gender (Male: Female)	4:5	3:1	1:4	0.099
Age (years) Median ± SD (range)	51 ± 14.4 (35–81)	52.5 ± 5.8 (45–59)	45 ± 19.7 (35–81)	0.730
BMI kg/m^2^ Mean ± SD	22.7 ± 6.4	22.8 ± 3.5	22.6 ± 8.5	1.000
Histology Moderately differentiated Poorly differentiated	7 (77.7%) 2 (22.2%)	3 (75%) 1 (25%)	4 (80%) 1 (20%)	0.858
Stage at triplet therapy 4A 4B 4C	4 (44.4%) 3 (33.3%) 2 (22.2%)	2 (50%) 1 (25%) 1 (25%)	2 (40%) 2 (40%) 1 (20%)	0.894
Involvement of ≥3 organs	4 (44.4%)	2 (50%)	2 (40%)	0.764
Liver metastasis	7 (77.7%)	3 (75%)	4 (75%)	0.858
Primary tumor resection Complete resection Partial or no resection	5 (55.5%) 4 (44.4%)	2 (50%) 2 (50%)	3 (60%) 2 (40%)	0.764
Baseline CEA > 5 μg/L	8 (88.8%)	3 (75%)	5 (100%)	0.236
Response Partial response Stable disease Progressive disease	1 (11.1%) 2 (22.2%) 6 (66.6%)	0 1 (25%) 3 (75%)	1 (20%) 1 (20%) 3 (60%)	0.638
Responder Non-responder	1 (11.1%) 8 (88.8%)	0 4 (100%)	1 (20%) 4 (80%)	0.343
Disease control rate	3 (33.3%)	1 (25%)	2 (40%)	0.635

SD, standard deviation; CEA, carcinoembryonic antigen; BMI: body mass index

**Table 2:** dMMR status, treatment responses, and survival of each patient with *BRAF*-mutated mCRC receiving triplet therapy.

	Age (Year) /Sex	Tumor Location	Primary Surgery	Metastasis Foci	dMMR	Best Response	PFS (Months)	OS (Months)
Patient 1	51, female	Right colon	No	Liver, lung, pancreas	ND	SD	6.9	7.5
Patient 2	45, female	Left colon	No	Liver, lung	No	PD	2.3	5.3
Patient 3	81, female	Left colon	R0 resection	Liver, lung	Yes	SD	19.3	19.3
Patient 4	41, male	Left colon	R0 resection	Liver, lung, adrenal gland, pancreas	No	PR	5.4	26.5
Patient 5	59, male	Right colon	R0 resection	Liver, peritoneum, pancreas	ND	PD	3.1	7.0
Patient 6	45, male	Right colon	R0 resection	Liver, peritoneum,	No	PD	1.5	1.5
Patient 7	54, male	Right colon	R1 resection	Peritoneum, Paraaortic lymph nodes	No	PD	2.6	2.6
Patient 8	35, female	Left colon	R0 resection	Peritoneum	Yes	PD	2.4	2.4
Patient 9	69, female	Left colon	No	Liver, lung, peritoneum, bone	ND	PD	3.0	9.2

dMMR, deficiency of mismatch repair genes; ND, not done; PFS, progression-free survival; OS, overall survival; SD, stable disease; PR, partial response; PD, progressive disease; mCRC: metastatic colorectal cancer; R0: complete resection in gross with microscopically negative surgical margin; R1: complete resection in gross with microscopically positive surgical margin.

**Table 3:** Adverse events in all patients receiving triplet therapy for *BRAF*-mutated mCRC.

Adverse Events	Grade 1–2 (%)	Grade 3 (%) †	Any Grade (%)
Anemia	1 (11.1)	0 (0)	1 (11.1)
Neutropenia	0 (0)	0 (0)	0 (0)
Thrombocytopenia	0 (0)	0 (0)	0 (0)
Fatigue	0 (0)	0 (0)	0 (0)
Nausea	2 (22.2)	2 (22.2)	4 (44.4)
Vomiting	3 (33.3)	0 (0)	3 (33.3)
Hair loss	1 (11.1)	0 (0)	1 (11.1)
Abnormal liver function	6 (66.6)	0 (0)	6 (66.6)
Acute kidney injury	2 (22.2)	0 (0)	2 (22.2)
Hypertension	4 (44.4)	2 (22.2)	6 (66.6)
Diarrhea	1 (11.1)	1 (11.1)	2 (22.2)
Paresthesia	2 (22.2)	0 (0)	2 (22.2)
Skin rash	4 (44.4)	0 (0)	4 (44.4)
Dermatitis	4 (44.4)	2 (22.2)	6 (66.6)
Blurred vision	1 (11.1)	0 (0)	1 (11.1)

†: No patient had grade 4 adverse event in the study.

## Data Availability

The datasets used and/or analysed during the current study are available from the corresponding author on reasonable request.
